# New Infirmary Scheme at Bristol: The Reorganisation of the Indoor Medical Work

**Published:** 1914-08-08

**Authors:** 


### NEW INFIRMARY SCHEME AT BRISTOL.

### The Reorganisation of the Indoor Medical Work.

The new infirmary buildings at Southmead are now approaching completion, and the Bristol Guardians will soon be in a position to start the scheme we described in our issue of April 25, 1914, for reorganising their indoor medical work. In this connection the history of Poor-Law administration in Bristol is of interest and illustrates the advantages to be derived from the amalgamation of Poor-Law Unions. Until 1897 there were three Boards of Guardians in that city, the area of two of them extending far outside its boundaries. In that year the municipality was enlarged, and one Poor-Law Union formed for the new area. Certain rural districts were, however, excluded, and a workhouse was built at Southmead to serve them. In 1904 a further extension of the city boundaries took place, involving a similar increase of the Bristol Poor-Law Union, and the Southmead Workhouse passed into the hands of the Bristol Guardians, who now found themselves in possession of three mixed workhouses situated at Southmead, Stapleton, and Eastville. Although each of these contained accommodation for sick patients no one of them satisfied the modern requirements for a hospital. Things were so bad, indeed, that the Local Government Board again and again urged upon the Guardians the necessity for the provision of better hospital buildings. After considering various schemes over a period of many years, the Guardians finally decided to construct a modern hospital at Southmead by adapting some of the old buildings and erecting new ones at a cost of about £50,000.

This reconstruction is now approaching completion. The main part of the hospital consists of seven pavilions connected by corridors. Five of the blocks are intended for cases of acute illness. Each block consists of two storeys, with thirty-six beds on each floor. Each ward unit consists of a large ward of thirty-two beds, with two small isolation rooms, one having three beds, the other one bed, a duty-room, day-room, linen-room, store-room, bathroom, and sanitary tower. The sixth block provides accommodation for fifty-four children, and is divided into a number of small wards so as to provide facilities for isolation and diminish the risk of infection. The seventh block is the maternity department, consisting of two wards of ten beds each; the intention being that one shall be in use whilst the other is undergoing disinfection. Separate from this block, but adjacent to it, is a pre-maternity department containing twelve beds for the reception of pregnant women awaiting confinement. Upon the top of this building it is proposed to build an operating theatre. Each of the blocks is provided with projecting balconies. Some of the old buildings have been adapted to receive convalescent patients and afford accommodation for sixty-eight beds, and a block originally intended for a fever hospital has been converted into wards for thirty phthisical patients. The Guardians propose to staff the institution with a medical superintendent, two assistant medical officers, a visiting physician and a visiting surgeon, a matron, and sixty nurses. We have already commented upon this staff.

If the medical officers' work were to be confined to Southmead it would compare favourably with the staffing of most Poor-Law infirmaries, but this is not the Guardians' intention. They suggest that one of the medical officers shall be resident at Stapleton, where accommodation is to be provided for at least 530 imbeciles and feeble senile cases. In addition, the ordinary medical work at Eastville, where 1,000 of the healthy aged and able-bodied are to be housed, is to be undertaken by the Southmead medical staff. The work is obviously far too much to be carried out efficiently by three medical men. Their whole time should be fully occupied at Southmead if the cases are to be properly treated. The patients at Stapleton will require the undivided attention of one medical man, and more if many young imbeciles are received and any attempt is made to classify, educate, and train them. As for Eastville, since it is not intended apparently to retain there any cases of serious illness, the work would be best entrusted to a visiting medical officer resident in the neighbourhood. The general supervision of the medical work in all three institutions and the duty of advising the Guardians on all medical matters in the Union should devolve upon the medical superintendent at Southmead, and this the Guardians suggest should be the case. That there is room for improvement in the medical work is shown by the fact that in these three institutions, with an average daily population of over 2,000, only thirty-seven operations were performed in 1913. With the concentration of acute cases at Southmead, the provision of an operating theatre, and the appointment of a visiting surgeon one may expect a great increase in the surgical work performed. It is to be hoped that the Guardians will reconsider the matter of their medical staff and will not allow their scheme to be spoilt, as so many Poor-Law schemes have been spoilt, by understaffing, nor let their buildings at Southmead become, what many admirably built Poor-Law infirmaries are, merely a home for the sick, but a hospital for their cure.

